# Can anti-TNFα Antibodies Affect SARS-CoV-2 Disease Outcomes?

**DOI:** 10.34172/apb.2022.066

**Published:** 2021-10-09

**Authors:** Mohammad Reza Khazdair

**Affiliations:** Cardiovascular Diseases Research Center, Birjand University of Medical Sciences, Birjand, Iran.

## Dear Editor,

The novel coronavirus (COVID-19) that first appeared in December 2019, subsequently named severe acute respiratory syndrome coronavirus type 2 (SARS-CoV-2) is rapidly spreading as a global pandemic. Following infection by SARS-CoV-2, systemic inflammatory response mediated by the release of large amounts of mediators including IL-6, IL-1b, TNFα and IL-2R in severe infected patients.^1,2^

It has been reported that severe COVID-19 infected patients had significantly higher serum levels of TNF than non-severe infected patients.^2^

In a case series study treatment of severe COVID-19 patients with infliximab (IFX), an anti-TNF antibody showed a rapid and temporary decrease in pro-inflammatory mediators such as IL-6 and other inflammatory markers (lactate dehydrogenase and C-reactive protein) along with clinical improvement in 6 of 7 infected patients. Lymphocyte count also increased in 5 patients after IFX treatment which was initially below (before IFX treatment). Moreover, 35% overall mortality at a similar stage of hospitalization was also observed in the 17 patients of the control group.^3^ In a study a man about 70 years old that treated with IFX (5 mg/kg) every 8 weeks and azathioprine for refractory ulcerative colitis infected with SARS-CoV-2, the symptoms of pneumonia were not observed on his chest computed tomography (CT). Also, some symptoms of this patient resolved within a few days without special treatment. But his wife, who was younger than that was not received immunosuppressive drugs, developed SARS-CoV-2 induced pneumonia.^4^

The results of a study showed that from 530 rheumatoid arthritis patients that treated with anti TNF agents (53.7%), 39.3% with other biologic disease-modifying drugs (bDMARDs) and treated with JAK inhibitors (7%) only 3 patients with mild COVID-19 were confirmed that managed at home without any complication in respiratory tract.^5^

In conclusion, TNF may exert pathogenic effects in coronavirus disease by augmenting the expression of angiotensin-converting enzyme 2 (ACE2) or by augmenting lymphopenia. Anti-TNF antibody by modulating of immune system and expression of ACE2 can useful for SARS-CoV-2 disease. But more clinical trials of anti-TNFα therapy for SARS-CoV-2 disease were suggested.

## Ethical Issues

Not applicable.

## Conflict of Interest

The author declares no conflicts of interest in this study.
